# Comprehensive Management of Polycystic Ovarian Syndrome and Secondary Infertility: Optimizing Fertility Outcomes

**DOI:** 10.7759/cureus.52597

**Published:** 2024-01-19

**Authors:** Shivani Khemani, Akash More, Nancy Nair, Namrata Choudhary, Jarul Shrivastava, Deepali Mamankar

**Affiliations:** 1 Clinical Embryology, Datta Meghe Institute of Higher Education and Research, Wardha, IND; 2 Obstetrics and Gynecology, Amardeep Hospital and Test Tube Baby Centre, Akola, IND

**Keywords:** gnrh long agonist protocol, gnrh short antagonist protocol, gnrh agonist (gnrh-a) trigger, ovarian stimulations, ovarian hyperstimulation syndrome (ohss), art, infertility, pcos

## Abstract

A 33-year-old woman with secondary infertility and polycystic ovarian syndrome (PCOS) is profiled in this case report. In 2020, an in-vitro fertilization/intracytoplasmic sperm injection pregnancy resulted in a missed abortion, which is part of the patient's medical history. In order to enhance fertility outcomes, this case report seeks to give an overall perspective on the treatment and medical care strategy for people with PCOS and previous cases of infertility. One of the physical characteristics of PCOS, bilateral polycystic ovaries, was seen in the patient. Treatments included long-term gonadotropin-releasing hormone agonist treatment, medications, and optimizing endometrial preparation. The patient was regularly monitored with routine ultrasound evaluations, hormone profiling, and psychological support. For patients with PCOS and secondary infertility, the case report emphasizes the value of individualized treatment strategies, close monitoring, and supportive care to enhance pregnancy outcomes. Clinicians handling situations similar to this one will greatly benefit from the conclusions and treatment plans offered in this case study, which emphasizes the need for a multifaceted strategy to deal with the complexity of PCOS-related infertility.

## Introduction

The most successful methods of treating infertility are in-vitro fertilization (IVF) and embryo transfer, which are utilized regularly globally. IVF is a multi-step treatment that begins with ovarian stimulation with gonadotropins and is followed by oocyte retrieval, laboratory fertilization, embryo culture, and the introduction of embryos into the uterus. The first step in maximizing the possibility of a healthy pregnancy is controlled ovarian stimulation, which is intended to induce the maturation of numerous oocytes [1].

The most prevalent endocrine problem in women of reproductive maturity is polycystic ovarian syndrome (PCOS), which is thought to be the main factor in instances of anovulation referred to in infertility centers. Following the failure of various methods of ovulation induction, women with PCOS can be provided IVF/intracytoplasmic sperm injection (ICSI) as a third-line treatment option, according to the current recommendations [2]. With an overall frequency of 5% to 15%, PCOS is the most prevalent endocrine condition in women and a leading cause of infertility. The patient must satisfy two out of the following three requirements: (1) oligo/anovulation; (2) analytic or biochemical symptoms of hyperandrogenism; and (3) polycystic ovaries, which have 12 or more follicles that are 2 to 9 mm in diameter, or enlarged ovarian volume, above 10 cm3 [3-4].

Generally, antagonist protocols are preferable for PCOS patients seeking ovarian stimulation since they guard against the ovarian hyperstimulation syndrome (OHSS) that typically occurs with agonist protocols [2-5]. Consequentially, we employed the antagonist treatment twice; however, the resulting embryos were of poor quality. We performed a third ovarian stimulation using an agonist protocol, followed by a frozen-thawed embryo transfer that resulted in successful implantation, and the couple is now the proud parents of twins. Long-agonist treatment, therefore, had a favorable effect on the PCOS patient, which is unusual and makes the study idiosyncratic.

## Case presentation

Patient information and chief complaint

This case report focuses on an Indian couple from the Vidarbha region of Maharashtra, India, who chose the Wardha Test Tube Baby Centre in Sawangi in order to achieve their desire to become parents despite being infertile. Secondary infertility has been the diagnosis of a 33-year-old woman for the last six years. The husband was a businessman and the wife was a homemaker; they were both descriptively counseled about all the procedures, merits, and demerits, and their informed consent was obtained. The chief complaints of the patient were an irregular menstrual cycle, excessive hair growth, and secondary infertility.

Personal and family history

The patient was initially diagnosed with PCOS in 2015. Since then, they have been trying for a baby, and in 2019, she had an IVF pregnancy but had a missed abortion at about nine weeks' gestation. At eight weeks and five days, the ultrasound revealed a fetal pole but no fetal movement, heart activity, or yolk sac. The husband's semen examination revealed that the motility was 85%, with a count of 135 million per ejaculation, which falls under the normal range as stated in the WHO guideline. The patient’s family had no history of infertility or any underlying condition related to infertility. The patient enrolled in our facility in 2021 in order to receive additional assessment and treatment.

Physical examination and hormonal profile

The patient appeared to be well-nourished and in no acute distress. The patient seemed to be a little bit overweight. Blood pressure, heart rate, and respiration rate all appear to be within the standard ranges. Table 1 shows the detailed hormonal profile of patients and indicates PCOS conditions.

**Table 1:** Hormonal profile

Parameters	Lab value	Reference value
Luteinizing hormone mIU/mL	16.3	2-15 mIU/mL
Testosterone ng/mL	1.3	0.15-0.7 ng/mL
Progesterone ng/mL	0.23	1-5 ng/mL
Estradiol pg/mL	31.87	30-400 pg/mL
Follicular stimulating hormone mIU/mL	3.07	3.5-12.5 mIU/mL
Anti-Mullerian hormone ng/mL	8.86	1.0-4.0 ng/mL
Prolactin ng/mL	15.0	2-29 ng/mL

Ultrasound findings

**Figure 1:**
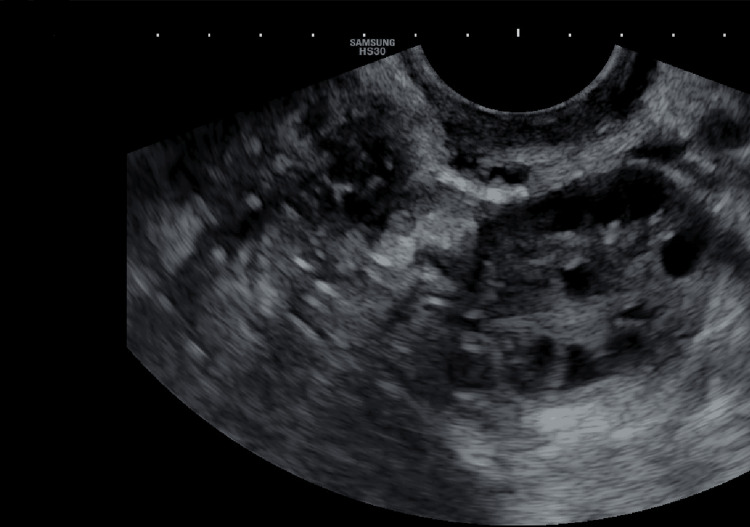
Transvaginal sonography image of the patient's left ovary on day two of the menstrual cycle

**Figure 2:**
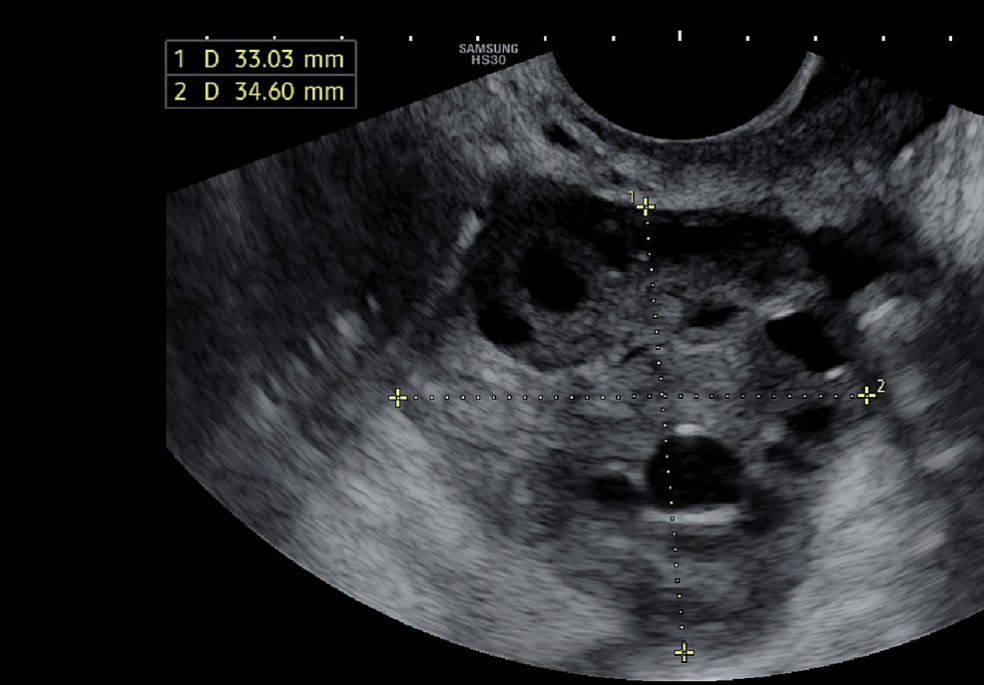
Transvaginal sonography image of the patient's right ovary on day two of the menstrual cycle

The uterus appears to be normal with normal echotexture. As seen in 2D of the patient's left and right ovaries, no dominant follicles were seen; the right ovary had 14 antral follicles, and the left ovary had 12 antral follicles (Figures 1-2).

Diagnosis and treatment

PCOS is the patient's diagnosis based on their medical history, clinical symptoms, and examinations. The patient's history of missed abortions after previous IVF/ICSI conception raises the possibility of PCOS-related infertility problems.

We chose the gonadotropin-releasing hormone (GnRH) short antagonist protocol for the first cycle of IVF/ICSI treatment as it is the widely accepted protocol for PCOS patients [2], along with 250 mg of metformin. However, in this case, we instructed the patient to take 300 mg of human menopausal gonadotropin (hMG) for seven days after taking 150 mg of follicle-stimulating hormone for three days. A human chorionic gonadotropin (HCG) injection of 10000 IU was given 36.5 hours prior to ovum pick-up (OPU) since it is responsible for oocyte maturation. After six days, just one day, five embryos, which were also of lower grade, were produced from poor-quality 2 MI and 5 MII oocytes that were retrieved. Despite the low quality of the embryo that had formed, it was nevertheless frozen, but no embryo transfer was carried out. After being counseled over the circumstances, the couple decided to proceed with the second round of IVF/ICSI treatment.

The GnRH short antagonist protocol with metformin was followed in the second cycle as well, with the exception that the dose of hMG was increased to 450 mg. The outcomes were the same as in the first cycle, with one poor-quality embryo formed and frozen on day five; no embryo transfer was carried out this time either. Following two unsuccessful cycles, the couple opted to proceed with a third IVF/ICSI cycle. This time, however, we chose to use the GnRH long agonist protocol and maintained the 250 mg metformin dose. Following OPU, 2 MI and 6 MII oocytes were obtained. Three days after the ICSI of 6 MII oocytes, two-day two embryos among the remaining six embryos were frozen, and two good-quality embryos among the remaining four embryos were frozen after another three days. Following OPU, platelet-rich plasma therapy was done using a transvaginal ultrasound scan to thicken the endometrium. The patient was then prescribed cabergoline (5 mg) twice daily for three days; along with it, a calcium gluconate intravenous infusion (three days) was also given, which prevents OHSS, and metformin (continued until embryo transfer). Oestradiol (2 mg twice daily) and 150 mg of progesterone were administered to the patient 20 days prior to the embryo transfer in order to increase endometrial receptivity. Following this, a sequential frozen-thawed embryo transfer was performed. The embryos were thawed using a widely recognized thawing kit, and two-day two and two-day five embryos were transferred.

Follow-up

Post-embryo transfer, the patient was advised to avoid any strenuous activity or heavy lifting and take proper rest, followed by Calcirol sachets for calcium intake, prednisolone (5 mg), progesterone, multivitamins, and iron supplements, and was asked to take a pregnancy test after 14 days. The urine pregnancy test tested positive, after which serum β-hCG was confirmed with a value of 250 mlU/ml, few weeks later, upon follow-up sonography, the fetus appeared to be a twin with a normal growth rate. The patient was advised to continue with the medications.

## Discussion

Around 70% to 80% of women experience infertility as a result of PCOS, as is evident from numerous papers, which include infertility as one of the major causes of PCOS [4,6-8]. Women with PCOS are susceptible to early pregnancy loss or first-trimester miscarriage, regardless of whether they conceive spontaneously or with IVF/ICSI. During her first IVF/ICSI cycle, our patient experienced early pregnancy loss, which affects between thirty percent and fifty percent of PCOS women in comparison with 10% to 15% of normal women [9-10].

Many studies comparing GnRH antagonists' short protocol and GnRH agonists' long protocol have found that GnRH antagonists are a superior alternative for PCOS patients because they suppress the luteinizing hormone surge, which shields them from developing OHSS; however, in nearly every trial, the rates of clinical pregnancy and live births were essentially the same [1-2,5,11-12]. Our patient conceived twin healthy baby girls by administering a long GnRH agonist protocol followed by a frozen-thawed embryo transfer before twice failing to conceive by administering a short GnRH antagonist protocol, in spite of studies suggesting better results with this approach. Fouda et al. evaluated the effectiveness of calcium infusion over cabergoline in the treatment of IVF patients who were at high risk for OHSS [13]. The extended GnRH agonist regimen was used to stimulate 180 individuals who were at high risk of having OHSS and were randomly assigned in a ratio of one to one to the cabergoline group and the calcium gluconate group, and it was discovered that calcium gluconate was more advantageous for preventing OHSS. There are other articles comparing calcium gluconate, cabergoline, and other medications for preventing OHSS [14], but based on our research, none of them demonstrated the combined effect of calcium gluconate and cabergoline, with the exception of one article that contrasted the combined effects of these two with calcium gluconate infusion alone and found that the combined effect was beneficial [15]. We made the decision to administer the patient a cabergoline tablet and a calcium gluconate (10%) intravenous infusion together (starting on the day of OPU, for three days) since OHSS is more severe in PCOS women receiving IVF/ICSI treatment because of increased arterial permeability, which causes proteinaceous fluid to flow into third spaces [16]. Renin secretion is one way that calcium infusion modulates the renin-angiotensin system. This has the effect of decreasing angiotensin II synthesis downstream, which lessens the stimulatory effect on the production of vascular endothelial growth factor. When high-risk patients, like those with PCOS, undergo assisted reproductive technology cycles, these changes in pathobiological mechanisms help prevent severe OHSS. This method is capable of reducing OHSS without having a significant negative impact [17].

## Conclusions

Following a long GnRH agonist protocol and taking the recommended medications resulted in the formation of four high-quality embryos, which later assisted in the conception of twins. As compared to other articles, our case has practiced a long GnRH agonist protocol, and it turned out to be a success. As for the medications given to the patient, cabergoline and calcium gluconate (10%), despite the threat of OHSS, they worked out to be a positive intervention for our patient.
